# A Differential Privacy Strategy Based on Local Features of Non-Gaussian Noise in Federated Learning

**DOI:** 10.3390/s22072424

**Published:** 2022-03-22

**Authors:** Xinyi Wang, Jincheng Wang, Xue Ma, Chenglin Wen

**Affiliations:** 1 School of Automation, Hangzhou Dianzi University, Hangzhou 310018, China; wxy840966221@163.com (X.W.); bigpaopaofishkk@163.com (J.W.); xuema1992@163.com (X.M.); 2 School of Automation, Guangdong University of Petrochemical Technology, Maoming 525000, China

**Keywords:** federated learning (FL), differential privacy, Kalman filter, non-Gaussian noise

## Abstract

As an emerging artificial intelligence technology, federated learning plays a significant role in privacy preservation in machine learning, although its main objective is to prevent peers from peeping data. However, attackers from the outside can steal metadata in transit and through data reconstruction or other techniques to obtain the original data, which poses a great threat to the security of the federated learning system. In this paper, we propose a differential privacy strategy including encryption and decryption methods based on local features of non-Gaussian noise, which aggregates the noisy metadata through a sequential Kalman filter in federated learning scenarios to increase the reliability of the federated learning method. We name the local features of non-Gaussian noise as the non-Gaussian noise fragments. Compared with the traditional methods, the proposed method shows stronger security performance for two reasons. Firstly, non-Gaussian noise fragments contain more complex statistics, making them more difficult for attackers to identify. Secondly, in order to obtain accurate statistical features, attackers must aggregate all of the noise fragments, which is very difficult due to the increasing number of clients. We conduct experiments that demonstrate that the proposed method can greatly enhanced the system’s security.

## 1. Introduction

With the rapid development of the Internet of things (IoT), an increasing number of devices are connected to the Internet [1,2], and the large amounts of data generated by these devices can be mined through machine learning and other artificial intelligence (AI) technologies to find value and improve the efficiency of production and operation [3,4,5,6,7]. Sometimes, training a decent machine learning model must require cooperation between multiple devices, and their data sets need to be shared with each other. However, most users are reluctant to share their data sets, as this involves private or other important information. Once they share the data, it is difficult for them to control it, which may lead not only to privacy leaks, but also to being threatened by malicious partners so as to cause serious damage [8,9], raising privacy concerns [10]. As a result, it is not feasible to train a decent model by directly sharing data, which results in “data islands”.

In order to solve the aforementioned problems, Google proposed federated learning (FL) [11], which transfers the data storage and model training phase to the clients (namely devices, including mobile phones, smart bracelets, pads and other terminals in the IoT), while the clients only upload metadata instead of the original data [12]. Metadata refers to the parameter information of a neural network, including its structure, gradient and weight. In this way, it can reduce communication pressure and improve data security. FL can share data value without sharing original data to mitigate the problem of “data islands”.

Although FL plays a significant role in privacy preservation, its main objective is to prevent peers from stealing data, as there is no protection ability against external attacks [13]. Research shows that malicious attackers can utilize reconstruction and other technologies to infer the original client data. Zhu et.al proposed an attack method [14] in which attackers can obtain original data through the differences in gradient information.

Scholars have tried encrypting metadata to defend against external attacks, for example by using homomorphic encryption and differential privacy methods. Even if malicious attackers steal the data during the transmission process, they cannot know the specific results of the actual data. For example, Phong proposed a deep learning system based on homomorphic encryption [15] to upload encrypted data to a cloud center. This system can effectively protect the privacy of gradient information. In federated learning, the communication cost is a major concern [16]. However, homomorphic encryption involves high computing and communication performance demands. It requires the original data to perform a large amount of the encryption operations and to transmit a large number of ciphertexts, which greatly increases the burden of the system.

Compared with the homomorphic encryption algorithm, differential privacy has been used because of its theory guarantee, simple algorithm and lower system performance requirements [17]. It can be easily implemented on small devices such as smart phones, which is very suitable for the application scenario of federated learning. Differential privacy was first proposed by Dwork in 2006 [18]. The basic approach is to add noise to the data so that the attacker cannot analyze the content of the original data through the data differences. McMahan applied differential privacy in federated learning to build a language prediction model [19] and achieved good results. Moreover, differential privacy has been applied in real life. Google (Mountain View, CA, USA) [20], Apple (Cupertino, CA, USA) [21], Microsoft (Washington, DC, USA) [22] and other companies have adopted differential privacy mechanism to collect user data for model training in a safe way.

In the differential privacy approach, random noise must be added to the data, mostly using the Laplace or Gaussian mechanism. Although adding noise with the Laplacian distribution or Gaussian distribution on the data has a certain protection ability, as its statistical properties are easy to identify, it still can be decrypted by experienced attackers. Attacks from the outside pose a great threat to the security of federated learning system and hinder the application of federated learning in IoT. Therefore, there is an urgent need to improve the security of data during transmission in FL systems.

## 2. Related Works

In order to improve the security of federated learning system, this paper proposes a federated learning differential privacy preservation strategy based on local features of non-Gaussian noise and aggregates the noisy metadata through a sequential Kalman filter (NGDP-FedSKF). We name the local features of non-Gaussian noise the non-Gaussian noise fragments. The basic approach is as follows. For a trained neural network, firstly the non-Gaussian noise is divided into several fragments, then one of the fragments is added to the metadata randomly and the noisy metadata are uploaded to the server. Secondly, the sequential Kalman filter is used in the server to aggregate the metadata for each client and to obtain a noisy global model, which consists of real metadata and added non-Gaussian noise. Thirdly, the noisy global model is sent to the client. Finally, in the client, a novel filter is designed to denoise and decrypt the noisy global model. The precision of the denoised global model is close to that achieved without adding noise. In this way, the training accuracy of federated learning system does not show significant decline and the ability to resist external attacks greatly enhanced, meaning the security of the system is significantly improved.

The main contributions of this paper are as follows: (1) we propose a differential privacy encryption strategy based on a class of non-Gaussian noise, making it difficult to decrypt the data using existing differential privacy decryption technology; (2) we divide a piece of random noise into multiple fragments, meaning we must aggregate all of the pieces of information into a whole in the server before we can design a method to decrypt it; (3) for the aggregated non-Gaussian noise information, we design a tailored filtering method to remove it, which has a good decryption effect on the existing encryption methods.

This paper is organized as follows. Section 1 provides a general introduction. Section 2 introduces the related work. Section 3 provides a detailed description of the proposed method. Section 4 provides a simulation and analysis of the experiments. Section 5 provides the conclusions and directions for future work.

## 3. Proposed Approach

### 3.1. Approach Overview

The main purpose of this subsection is to introduce the main process of the proposed NGDP-FedSKF method.

In this paper, all edge devices are treated as clients, and we set up a trusted client server called the server. We refer to the collection of clients and the server as a cluster.

Although federated learning is a good solution to the problem of privacy preservation within a cluster from the point of view of system stability, the following problems still remain:

- Delay and packet loss during data transmission [23];
- Inadequate defense against external attacks [24].

**Figure 1:**
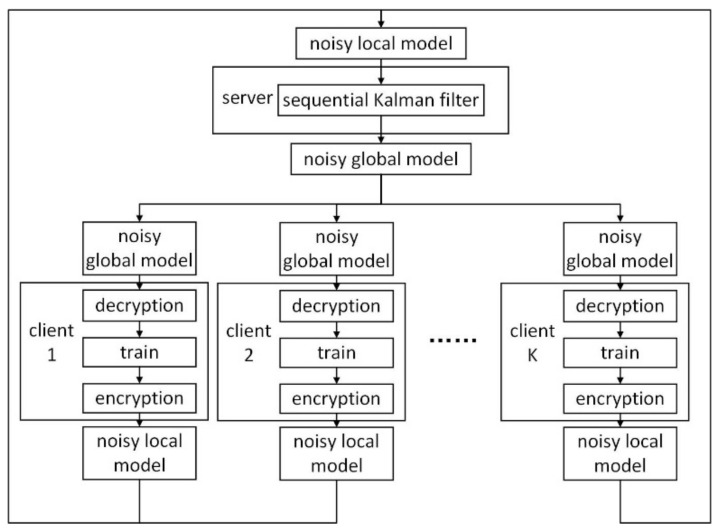
Description of the federated learning structure.

We propose a differential privacy strategy based on local features of non-Gaussian noise and aggregate the metadata from each client with a sequential Kalman filter in the server, which greatly improves the security of the data transmission and allows real-time updates. Once the metadata reach the server, they can be aggregated immediately if the server is in an idle state. As depicted in Figure 1, the proposed method is as follows:

1. At first, the server initializes a global model and sends its structure and initialized metadata to each client for training, where the metadata includes the connection weight and bias of the global model;
2. If it is not the first round, each client denoises and decrypts the noisy metadata from the global model issued by the server with its secret key and takes the result as the initial value of this training round;
3. After the training process, each client adds a non-Gaussian noise fragment with a non-zero mean value to the metadata randomly, then uploads it to the server. Based on the noise fragment, the client will generate a secret key and save it locally;
4. The server aggregates the noisy metadata with a sequential Kalman filtering algorithm and sends the noisy metadata from the global model to the clients;
5. Steps 2 to 4 are repeated until reaching satisfactory testing performance.

Given a fixed set of K clients, client l has a fixed local data set $P_{l}$ with $\left|P_{l}\right|$ samples. The m clients are picked in each round to participate in training. $\left|P\right|=\sum_{l=1}^{m}\left|P_{l}\right|$ is the total number of samples in a round. Our goal is to minimize the loss function f(ω):

(1) $$\min f\left(\omega \right)$$

(2) $$f\left(\omega \right)=\sum_{i=1l}^{m}\frac{\left|P_{l}\right|}{\left|P\right|}f_{l}\left(\omega \right)$$

where $f_{l}(\omega )$ is the loss function of the client l, l=1,2,⋯,m. The simplified pseudo-code for the NGDP-FedSKF is illustrated in Algorithm 1.
**Algorithm 1**: NGDP-FedSKF01.m clients participate in the training in each round;02.α is metadata(model parameter);03.β is non-Gaussian noise randomly added by each client.04.**For server executes:**05.** Input:**$x_{t}^{1},x_{t}^{2},\ldots ,x_{t}^{m}$ where $x_{t}^{l}=\alpha_{t}^{l}+\beta_{t}^{l}$,  l=1,2,⋯m
06. **Output:**
$x_{t+1}$07.  initialize $\alpha_{0}$
08.   **for** each round t=1,2,…
**do**09.   **for** each client l=1,2,⋯m
**do**10.    
$x_{t+1}^{l}\leftarrow SKF(x_{t}^{l})$
11.   **end for**12.   $x_{t+1}=x_{t+1}^{m}$
13.  **end for**14.
15.**For client executes:**16. **Input:**$x_{t}^{}$
17. **Output:**
$x_{t}^{1},x_{t}^{2},\ldots ,x_{t}^{m}$
**where**
$x_{t}^{l}=\alpha_{t}^{l}+\beta_{t}^{l}$, l=1,2,⋯m18.  **for** each round t=1,2,…
**do**19.   **for** each client l=1,2,⋯m
**do**20.    $\alpha_{t}^{l}\leftarrow$ decrypt $x_{t}$
21.    $\alpha_{t}^{l}\leftarrow \alpha_{t}^{l}$ Update by stochastic gradient descent22.   
$x_{t}^{l}\leftarrow \alpha_{t}^{l}+\beta_{t}^{l}$
23.   **end for**24. **end for**

**Remark** **1.** *This section introduces the overall process of the proposed method, in which the noise adding (encryption) method, SKF algorithm and decryption method are in Section 3.2, Section 3.3 and Section 3.4, respectively.*

### 3.2. Noise-Adding Method Based on Non-Gaussian Fragments

The main purpose of this subsection is to give an outline of differential privacy technology and present the noise-adding method based on non-Gaussian fragments we proposed.

The definition of differential privacy was first proposed by Dwork. Let data sets D and $D^{\prime }$ differ on at most one element, where Φ is a random algorithm. For any output S⊂Range(Φ), if Equation (3) is true, then algorithm Φ satisfies (ε,δ) differential privacy:

(3) $$\mathrm{Pr}\left[\Phi (D)\in S\right]\leq \mathrm{Pr}\left[\Phi (D^{\prime })\in S\right]\times e^{\varepsilon }$$

where ε is the privacy budget and δ is the failure probability.

The sensitivity L(f) can measure the output variation of the function f over two data sets D and $D^{\prime }$. If L(f) is very large, subtle changes in the data set can lead to significant output differences. According to different calculation methods, sensitivity L(f) can be defined as sensitivity $L_{1}(f)$ and sensitivity $L_{2}(f)$ as follows:

(4) $$L_{1}(f)=\max{\left\|f(D)-f(D^{\prime })\right\|}_{1}$$

(5) $$L_{2}(f)=\max{\left\|f(D)-f(D^{\prime })\right\|}_{2}$$

Differential privacy can be implemented in many ways. At present, the main method is to add random noise disturbance. For a row data set α, the encrypted data set is α+β, where β is random noise. For the Laplace mechanism, if the random noise follows the Laplace distribution $Laplace(0,\frac{L_{1}(f)}{\varepsilon })$, it can satisfy ε-Differential privacy. For the Gaussian mechanism, if the random noise follows the Gaussian distribution $Gaussian(0,\sqrt{2\ln\frac{1.25}{\delta }}\times \frac{L(f_{2})}{\varepsilon })$, it can satisfy (ε,δ)-Differential privacy.

Differential privacy technology has been used in machine learning. For example, Geyer proposed a user-level differential privacy federated learning framework [25], which provides differential privacy preservation for users. Compared with other encryption algorithms, differential privacy is very suitable for federated learning due to its low implementation cost. However, for the Laplace mechanism and Gaussian mechanism, their simple statistical features can still be decrypted by experienced attackers. For example, when the mean value is zero, it can be removed easily by using an exponential filter, so the security needs to be strengthened. Therefore, we propose a differential privacy strategy based on non-Gaussian noise fragments.

The non-Gaussian noise β with a non-zero mean value has the distribution of p(x), where a<x<b; m clients are picked at each round to participate in training. As shown in Equation (6), β is divided into r⋅m equal parts and $\beta_{i}$ has the distribution of $p_{i}(x)$, where r≥1, 1≤i≤rm, while the range of values of x show in Equation (7):

(6) $$\beta =\beta_{1}\cup \beta_{2}\cup \cdots \cup \beta_{rm}$$

(7) $$\frac{\left(b-a)(i-1\right)}{rm}\leq x\leq \frac{(b-a)i}{rm}$$

A selection matrix $\Gamma^{l}$ produced by client l can determine which fragment will be added on $\alpha^{l}$, as shown in Equation (8):

(8) $$\beta^{l}\sim \Gamma^{l}p(x)$$

Then, the noise $\beta^{l}$ will be added to the metadata $\alpha^{l}$ and the noisy metadata can be represented as $x^{l}$, $x^{l}=\alpha^{l}+\beta^{l}$. Finally, $x^{l}$ will be uploaded to the server as a local model parameter for sharing.

**Remark** **2.** *This subsection introduces the noise-adding method based on non-Gaussian fragments. Compared with the traditional methods, the proposed method has stronger security performance for two reasons. First, the noise we add has more complex statistics and it is more difficult for attackers to identify. Second, in order to obtain accurate statistical features, one must aggregate all the noise fragments. As the number of clients increases, it becomes less and less possible to intercept all of the fragments.*

### 3.3. Sequential Kalman Aggregation Algorithm

The main purpose of this subsection is to elaborate the sequential Kalman aggregation algorithm in the case of additive noise. In NGDP-FedSKF, we utilize it to aggregate the noisy metadata that come from clients.

The federated averaging algorithm (FedAvg) is the baseline FL aggregation algorithm [26]. However, the delay and packet loss of updates during communication are ignored [27]. In practice, it cannot aggregate the local models’ parameters until all of them arrive at the server, which results in poor reliability and controllability. In this paper, we apply a sequential Kalman filter (SKF) to aggregate the local models’ noisy metadata [28] in real-time in the order of arrival. This approach is improved on the basis of a classical Kalman filtering algorithm to adapt to the random arrival of parameters, which is very suitable for the application scenario of federated learning.

In order to update the parameters online via sequential Kalman filter, the model needs to establish the state equation and measurement equation according to the Kalman filter [29,30,31,32,33]:

We can denote the status value of the k period in client l as $\alpha^{\left(l\right)}(k)$. After adding the noise fragment $\beta^{\left(l\right)}(k)$, the new status value is updated to $x^{\left(l\right)}(k)$ via the state equation shown in Equation (9):

(9) $$x^{\left(l\right)}(k)=C_{1}\alpha^{\left(l\right)}(k)+C_{2}\beta^{\left(l\right)}(k)$$

where l=1,2⋯,m; $C_{1}$ and $C_{2}$ are regulatory factors.

Considering the dynamic relationship between the k and k+1 periods, the state models shown in Equation (10) involves the concept of random walks:

(10) $$x^{l}(k+1)=Ax^{l}(k)+w(k)$$

where A is the state transition matrix; w(k) is process noise, which is Gaussian white noise with a mean of zero, the variance of which is Q(k) and Q(k)≥0.

The measurement equation is updated as Equation (11):

(11) $$y^{l}(k+1)=C_{1}\alpha^{\left(l\right)}(k+1)+C_{2}\beta^{\left(l\right)}(k+1)+v(k+1)=Hx^{l}(k+1)+v(k+1)$$

where H is the measurement matrix; v(k+1) is measurement noise, which is Gaussian white noise with a mean of zero, the variance of which is R(k+1) and R(k+1)≥0.

We can set $x^{0}(k\left|k\right.)$ as the random initial value of the global model as in Equation (12):

(12) $$x^{0}(k\left|k\right.)=\alpha (0)$$

The sequential Kalman filter update process is as follows:

(13) $${\hat{x}}^{1}(k+1\left|k\right.)=Ax^{0}(k\left|k\right.)$$

(14) $$P^{1}(k+1\left|k\right.)=AP^{0}(k\left|k\right.)A^{T}+Q(k)$$

(15) $$K^{1}(k+1)=P^{1}(k+1\left|k\right.)H^{T}{\left[HP^{1}(k+1\left|k\right.)H^{T}+R(k+1)\right]}^{-1}$$

(16) $${\hat{x}}^{1}(k+1\left|k+1\right.)={\hat{x}}^{1}(k+1\left|k\right.)+K^{1}(k+1)[y^{1}(k+1)-H{\hat{x}}^{1}(k+1|k)]$$

(17) $$P^{1}(k+1\left|k\right.+1)=[I-K^{1}(k+1)H]P^{1}(k+1\left|k\right.)$$

⋮

(18) $$K^{m}(k+1)=P^{m-1}(k+1\left|k+1\right.)H^{T}{\left[HP^{m-1}(k+1\left|k\right.+1)H^{T}+R(k+1)\right]}^{-1}$$

(19) $${\hat{x}}^{m}(k+1\left|k+1\right.)={\hat{x}}^{m-1}(k+1\left|k\right.+1)+K^{m}(k+1)[y^{m}(k+1)-H{\hat{x}}^{m-1}(k+1|k+1)]$$

(20) $$P^{m}(k+1\left|k\right.+1)=[I-K^{m}(k+1)H]P^{m-1}(k+1\left|k\right.+1)$$

(21) $$\hat{x}(k+1\left|k+1\right.)={\hat{x}}^{m}(k+1\left|k+1\right.)$$

(22) $$P(k+1\left|k\right.+1)=P^{m}(k+1\left|k\right.+1)$$

where $\hat{x}(k+1\left|k+1\right.)$ is the global model’s noisy metadata for the new round.

(23) $$\hat{x}(k+1|k+1)={\hat{x}}^{m}(k+1|k+1)=E\{x^{m}(k+1|k+1)|{\hat{x}}^{m-1}(k+1|k+1),y^{1}(k+1),y^{2}(k+1),\cdots ,y^{m}(k+1)\}$$

**Remark** **3.** *Through the method proposed in this subsection, the server can asynchronously update the global model in real time in the case of additive noise, and can achieve similar or even better results than using centralized filtering.*

### 3.4. Noise Elimination Method

The main purpose of this subsection is to introduce the decryption method for the clients. In order to obtain high-precision data, the clients must eliminate the noise after the noisy global model arrives.

The clients obtain the noisy global model parameter $\hat{x}(k+1\left|k+1\right.)$ from the server, which involves the joint estimation of the global model parameter α(k+1) and noise β(k+1). The clients have the distribution information for β(k+1), which has the ability to eliminate as much noise as possible by converting noise to white noise [34], as in Equation (24):

(24) $$\beta (k+1)=C_{\beta }\beta (k)+\eta (k)$$

where {η(k),k≥0} is white noise and its variance is $Q_{\eta }(k)$.

To design a new filter to remove the added noise, the new state value is G(k) in Equation (25). We need to establish the state equation and measurement equation as Equations (26) and (27), respectively:

(25) $$G(k+1)=\left[\begin{array}{c} \alpha (k+1)\\ \beta (k+1) \end{array}\right]$$

(26) $$G(k+1)=A_{G}G(k)+\eta (k)$$

(27) $$\begin{array}{l} y_{G}(k+1)=\hat{x}(k+1\left|k+1\right.)\\ \;=C_{1}\alpha (k+1)+C_{2}\beta (k+1)+\eta (k+1)\\ \;=\left[\begin{array}{cc} C_{1} & O\\ O & C_{2}C_{\beta } \end{array}\right]\left[\begin{array}{c} \alpha (k+1)\\ \beta (k+1) \end{array}\right]+\eta (k+1)\\ \;=\left[\begin{array}{cc} C_{1} & O\\ O & C_{2}C_{\beta } \end{array}\right]G(k+1)+\eta (k+1)\\ \;=H_{G}G(k+1)+\eta (k+1) \end{array}$$

The Kalman filtering process is as follows:

(28) $$\bar{G}(k+1|k)=A_{G}G(k|k)$$

(29) $$P_{G}(k+1|k)=A_{G}P(k|k)A_{G}^{T}+Q_{\eta }(k)$$

(30) $$K_{G}(k+1)=P_{G}(k+1|k)H_{G}^{T}{\left[H_{G}P_{G}(k+1|k)H_{G}^{T}+Q_{\eta }(k)\right]}^{-1}$$

(31) $$\bar{G}(k+1|k+1)=\bar{G}(k+1|k)+K_{G}(k+1)[y_{G}(k+1)-H_{G}\bar{G}(k+1|k)]$$

(32) $$P_{G}(k+1|k+1)=[I-K_{G}(k+1)H_{G}]P_{G}(k+1|k)$$

According to optimal estimation $\bar{G}(k+1\left|k+1\right.)$, we apply the selection matrix $U=\left[\begin{array}{cc} 1 & 0 \end{array}\right]$ to obtain the optimal estimation value $\bar{\alpha }(k+1)$ as the initial value α(k+1) of new a round, as Equations (33) and (34). After removing the noise, the precision of the model can be greatly improved.

(33) $$\left[\begin{array}{cc} 1 & 0 \end{array}\right]\bar{G}(k+1|k+1)=\left[\begin{array}{cc} 1 & 0 \end{array}\right]\left[\begin{array}{c} \bar{\alpha }(k+1)\\ \bar{\beta }(k+1) \end{array}\right]\alpha (k+1)=\bar{\alpha }(k+1)$$

(34) $$\alpha (k+1)=\bar{\alpha }(k+1)$$

**Remark** **4.** *Because clients have the statistical properties of the noise and decryption methods, they can design a filter to remove noise to obtain the optimal value. However, the attackers do not have prior knowledge, meaning they cannot effectively erase noise. Even if they utilize traditional Gaussian white noise filtering methods, the result they can obtain is not as accurate as the clients’ result or is even worse than using no decryption.*

## 4. Experiment Simulation

### 4.1. Data Set Preparation

In this paper, a rolling bearing data set from Case Western Reserve University (CWRU) is used for simulation tests. We use a part of the data set of one horsepower for a simulation test. A total of 1800 data samples are selected from the training set and 900 data samples are selected from the test set. Five dimensions are extracted through the pre-processing method to facilitate testing, and 9 fault types are generated by EDM.

### 4.2. Experimental Setting

The framework structure of the cluster is a server and four clients. The functional architecture of the system is shown in Figure 1. In order to simulate delays in the communication process in reality, we set the order of parameters arriving at the server as random; that is, the aggregation order of the SKF is random.

The sample number of the training set per client is 450, and 900 test samples are used to test the accuracy of each client’s model to obtain the average accuracy.

In this experiment, 5 layers of neural network are set, with 5, 21, 43, 25 and 9 nodes in each layer, respectively. The number of communication rounds is set as 50, and the number of neural network training epochs in the client is set as 50. We apply a stochastic gradient descent to train the local model.

In this experiment, we compare the accuracy levels and training times of FedAvg and FedSKF in adding non-Gaussian noise fragments with different mean values, which we name NGDP-FedAvg and NGDP-FedSKF, respectively. In order to verify the significant effects of encryption, we set up a test without decryption (not-decrypt). In this test, we apply FedSKF to aggregate the metadata, but unlike the NGDP-FedSKF experiment, in the final communication round, once clients receive the noisy global model, they calculate the accuracy immediately without decrypting the model. In addition, we set up another test (Gaussian decrypt) where we suppose that the attacker learns the statistical properties of the added noise and utilizes a Gaussian mechanism to denoise it.

**Figure 2:**
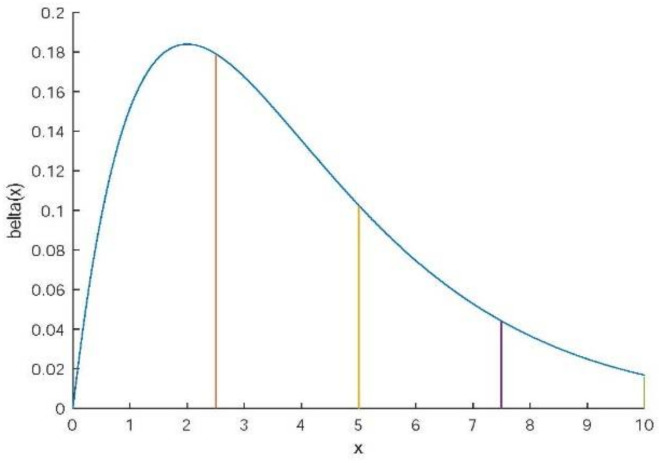
The chi-square distribution (4) was divided into four equal parts of 0≤x<2.5, 2.5≤x<5, 5≤x<7.5 and 7.5≤x<10.

The noise β we add has a chi-square distribution with a parameter of 4, while the range is from 0 to 10, as shown in Figure 2. We set coefficient ζ to change the mean value of the noise as Equation (34). In this experiment, we set ζ=0.2, ζ=0.4, ζ=0.6 and ζ=0 for the case with no added noise:

(35) $$\beta (x)\sim \zeta \chi^{2}\left(4\right)$$

### 4.3. Rresult Analysis

**Figure 3:**
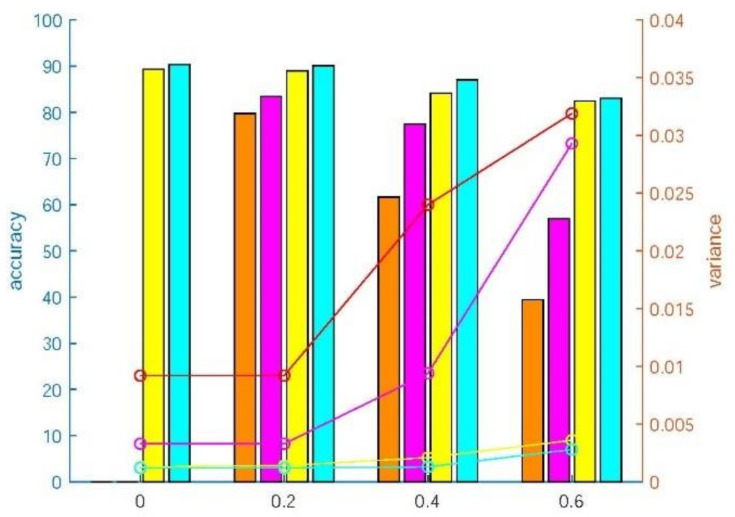
Comparison of the results for the different methods. Dark orange—not-decrypt; magenta—Gaussian decrypt; yellow—NGDP-FedAvg; cyan—NGDP-FedSKF. The line chart represents the variance values and the bar chart represents average accuracy values.

Applying the not-decrypt, Gaussian decrypt, NGDP-FedAvg and NGDP-FedSKF methods for training, we add different means of noise to the metadata. We repeat the experiments one hundred times. The accuracy levels in fault diagnosis are shown in Table 1 and the variance analysis results are shown in Table 2. The comparison of the results for the different methods is shown in Figure 3.

In terms of the accuracy shown in Table 1, NGDP-FedSKF shows better performance than NGDP-FedAvg, with the percentage being over 1%, 1.11%, 2.88% and 0.61%. Adding noise with different mean values will decrease the accuracy to different degrees. However, regardless of the aggregation method we use, the accuracy is much more than in the not-decrypt and Gaussian decrypt tests.

In terms of the stability shown in Table 2, the variance increases with the mean value of the added noise. NGDP-FedSKF shows better performance than NGDP-FedAvg as well. When a small amount of noise is added, the variance increase is not obvious. However, when ζ≥0.4, the variance increases rapidly.

As ζ=0.2, the accuracy is almost the same as without the noise; moreover, the security is improved. As ζ=0.4, although the accuracy is slightly decreased, the security is greatly improved. Therefore, ζ should not be too large or too small. If it is too large, it will have a great impact on the accuracy of the model and lead to a great decline in accuracy. If it is too small, it cannot achieve the effect of privacy protection.

The average training times for NGDP-FedAvg and NGDP-FedSKF are 35.3 s and 38.9 s respectively. Because the SKF algorithm is more complex than the federated average algorithm, the training time for NGDP-FedSKF is slightly higher than for NGDP-FedAvg, although this is acceptable.

Therefore, in terms of confidentiality, the accuracy of the not-decrypt case is significantly lower than the other cases. Even if the attackers learn the statistical properties of the added noise and utilize a Gaussian mechanism to denoise it, the accuracy will still be much lower than for NGDP-FedAvg and NGDP-FedSKF. This proves that the encryption method we have proposed has a significant protective effect.

### 4.4. Result Analysis

In terms of the accuracy and stability during fault diagnosis, the method proposed in this paper has a good privacy protection effect, because the accuracy of diagnosis for each client is significantly higher than in the not-decrypt case. This section analyzes the distances between different model parameters to further prove the effectiveness of the proposed methods from a theoretical perspective.

In a communication round, we set the parameter before encryption as α and the parameter after encryption through the proposed method as $\alpha_{1}$. Using the proposed method to decrypt $\alpha_{1}$, we can get ${\hat{\alpha }}_{1}$. We use the traditional Gaussian decryption method to decrypt $\alpha_{1}$ so as to get ${\hat{\alpha }}_{1}^{\prime }$.

The Euclidean distance is used here to calculate the distance between parameters for each model. The distance between α and $\alpha_{1}$ is ${\left\|\alpha -\alpha_{1}\right\|}_{2}$, which can be used to measure the encryption effect. The farther the distance is, the better the encryption effect and the less information is disclosed after being intercepted. The distance between α and ${\hat{\alpha }}_{1}$ is ${\left\|\alpha -{\hat{\alpha }}_{1}\right\|}_{2}$, which can be used to measure the decryption effect. The closer the distance, the better the decryption effect, meaning the client can obtain more accurate decryption results. The distance between α and ${\hat{\alpha }}_{1}^{\prime }$ is ${\left\|\alpha -{\hat{\alpha }}_{1}^{\prime }\right\|}_{2}$, which indicates the decryption effect of traditional Gaussian methods after being intercepted by external attackers. The farther the distance, the worse the decryption effect. After multiple tests and averaging of the results, ${\left\|\alpha -\alpha_{1}\right\|}_{2}=1.86$, ${\left\|\alpha -{\hat{\alpha }}_{1}\right\|}_{2}=0.2$, ${\left\|\alpha -{\hat{\alpha }}_{1}^{\prime }\right\|}_{2}=1.57$. This shows that the proposed method exhibits good security performance and does not have a great impact on the accuracy of the model. Even if external attackers use the traditional Gaussian method for decryption, they cannot accurately obtain the original data.

## 5. Conclusions and Future

In this work, we proposed a differential privacy strategy (NGDP-FedSKF) based on local features of non-Gaussian noise and aggregates of the noisy metadata through a sequential Kalman filter in federated learning scenarios to improve the security of the federated learning system. An encryption technique based on local non-Gaussian features was proposed to implement differential privacy. A data aggregation technique based on sequential filter in the center was designed to aggregate the models of each client online. A novel filter in the client was designed to decrypt the noisy aggregated metadata with non-Gaussian statistical characteristics. The method proposed here was proven using experiments, showing that in circumstances of appropriate noise, although the accuracy slightly decreased, the safety performance of the federated learning system was greatly improved. Moreover, it can aggregate local models’ noisy metadata online, solving the problems of delay and packet loss during data transmission. We suggest that the NGDP-FedSKF model is a suitable method to improve the defense capability of the federal learning system against external attacks.

There are still several points worthy of researching and improving in the future. One of the most important points is that the added noise should not be too large or small, since improving the privacy protection requires a loss of model accuracy. Therefore, we will consider searching for a more suitable mean of the noise to achieve the best balance between the privacy protection and precision of the model [35].

## Figures and Tables

**Table 1:** The fault diagnosis accuracy results.

Method	Diagnosis Accuracy (Unit: Percentage %)			
	ζ=0	ζ=0.2	ζ=0.4	ζ=0.6
Not-decrypt	-	79.75	61.67	39.48
Gaussian-decrypt	-	83.44	77.42	56.98
NGDP-FedAvg	89.33	89.00	84.15	82.44
NGDP-FedSKF	90.33	90.11	87.03	83.05

**Table 2:** The variance analysis results for the fault diagnosis accuracy.

Method	Accuracy Variance			
	ζ=0	ζ=0.2	ζ=0.4	ζ=0.6
Not-decrypt	-	0.0092	0.0240	0.0319
Gaussian-decrypt	-	0.0033	0.0093	0.0293
NGDP-FedAvg	0.0013	0.0014	0.0021	0.0036
NGDP-FedSKF	0.0012	0.0012	0.0013	0.0028

## Data Availability

Available online: https://engineering.case.edu/bearingdatacenter/welcome (accessed on 1 February 2022).

## Nomenclature

K	total number of clients
m	the number of picked clients in each round
l	a client
P	client data set
$\left|P\right|$	the number of samples of the client data set
f(ω)	loss function
α	metadata
β	non-Gaussian noise
x	metadata after adding noise
D	data set
Φ	random algorithm
Pr(⋅)	probability
ε	privacy budget
δ	failure probability
L(f)	sensitivity
p(⋅)	probability density function
Γ	selection matrix in encryption process
w	process noise
v	measurement noise
C	regulatory factors
k	time step
H	measurement matrix
A	state-transition matrix
$\hat{x}(k|k)$	state estimate
$\hat{x}(k+1|k)$	state prediction value
P(k+1|k)	state prediction error covariance matrix
P(k+1|k+1)	estimate error covariance matrix
K	Kalman gain matrix
$\tilde{\varphi }(k+1)$	prediction error
U	selection matrix in decryption process
ζ	coefficient of mean regulation
